# Astroblastoma – a case report of a rare neuroepithelial tumor with complete remission after chemotherapy 

**DOI:** 10.5414/NP300411

**Published:** 2011-10-18

**Authors:** M. Bergkåsa, S. Sundstrøm, S. Gulati, S.H. Torp

**Affiliations:** 1 Department of Laboratory Medicine, Children’s and Women’s Health, Faculty of Medicine, Norwegian University of Science and Technology (NTNU),; 2 Department of Oncology,; 3 Department of Neurosurgery,; 4 Department of Pathology and Medical Genetics, Faculty of Medicine, Norwegian University of Science and Technology (NTNU), St. Olavs University Hospital, Trondheim, Norway

**Keywords:** brain neoplasm, chemotherapy, glioma, immunohistochemistry, radiotherapy

## Abstract

Introduction: Astroblastoma is a rare glial tumor of uncertain origin affecting mostly children, adolescents and young adults. Given the rarity and the definitional problems concerning this tumor entity, the prognosis and appropriate treatment are at this point unclear. Case report: A 50-year-old Caucasian female presented with a seizure. Radiological findings showed a well-defined circumscribed tumor located in the right cerebral frontal lobe. The patient underwent primary surgery followed by postoperative radiotherapy. After 6 months the tumor recurred with multiple small lesions not available for surgery. Chemotherapy was administered with complete radiological response. Seven years after surgery and more than 6 years after completed chemotherapy the patient is free of disease. Histopathology revealed a gliomatous tumor with gemistocyte-like tumor cells arranged in palisades or strings and areas with perivascular pseudorosettes, consistent with astroblastoma. Immunophenotype and ultrastructural findings confirmed the diagnosis and verified the neuroepithelial origin. Conclusion: Astroblastomas are rare brain tumors and pose a challenge in the diagnostic and clinical approach. In general, they have an unpredictable course with a tendency of recurrence. This and other case reports support a survival benefit of chemotherapy, suggesting this as an important treatment option for these patients.

## Introduction 

Astroblastoma is a rare and controversial glial tumor first described by Bailey et al. [1, 2]. It is now, however, considered as a definite glioma entity of astrocytic origin [3, 4]. This tumor is mainly located to the cerebral hemispheres and affects mostly children, adolescents and young adults, however, congenital forms and astroblastomas in patients above 50 years of age have been reported as well [5, 6, 7, 8, 9, 10, 11, 12, 13]. 

Astroblastomas can be graded as either low- or high-grade (anaplastic/malignant) variant [3, 4]. Well-differentiated surgically accessible forms are associated with less frequent recurrence and prolonged survival. Still, the clinical course is unpredictable, since low-grade tumors may relapse comparable with anaplastic variants [3, 4]. 

## Case report 

**Figure 1.**
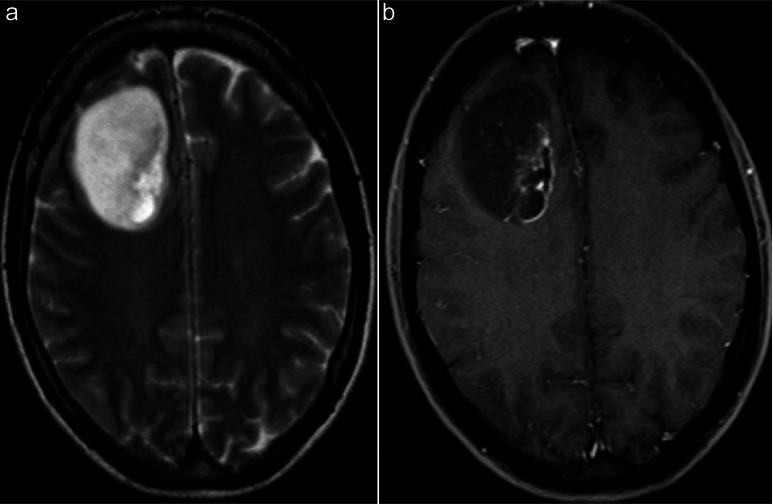
The primary tumor is well circumscribed with patchy contrast enhancement in the right frontal lobe. T2-weighted (a) and post-contrast T1-weighted (b) images.

A 50-year-old woman was admitted to hospital after a seizure. No neurologic deficits were recognized. Non-contrast enhanced computed tomography (CT) in September 2003 demonstrated a tumor-suspect lesion in the right frontal lobe surrounded by edema without midline extension. Magnetic resonance imaging (MRI) taken 4 days later showed a well defined, patchy contrast enhancing tumor measuring 6 × 3.5 × 5 cm with compression of the right lateral ventricle (Figure 1). Preoperative MRI data were imported into an ultrasound-based navigation system and used for surgical planning and resection guidance [14]. In the end of September 2003 a frontoparietal craniotomy was performed under general anesthesia, with the patient’s head resting in a Mayfield frame system attached to a reference frame for neuronavigation. Central parts of the tumor could easily be distinguished and appeared opaque. The Cavitron ultrasonic aspirator (CUSA) was applied to fragment and aspirate the tumor. Updated intra-operative ultrasound volumes were acquired during surgery and clearly showed tumor margins. The patient had no surgery-related neurological deficits, and a postoperative MRI scan showed gross total tumor resection. Histology was at first interpreted as anaplastic astrocytoma; however, final diagnosis was consistent with anaplastic astroblastoma. She received postoperative conformal 3D radiation therapy to a total dose of 54 Gy in 30 fractions; the last fraction was administered in December 2003. 

**Figure 2.**
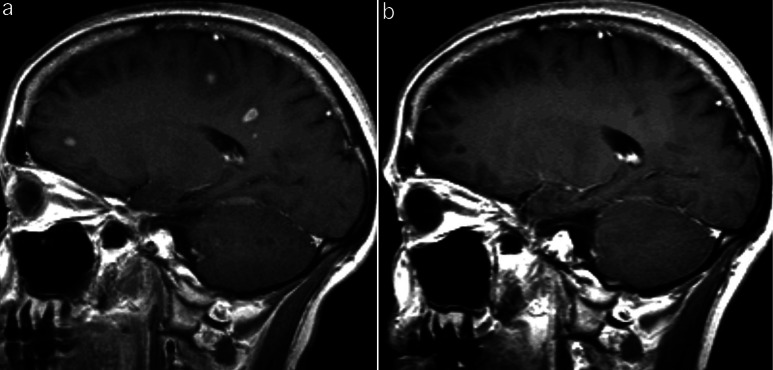
Multiple lesions suspected of multifocal tumor recurrence before (a) and after (b) chemotherapy. T1-weighted post-contrast images.

In March 2004 a scheduled MRI examination showed multiple ring enhancing lesions in both hemispheres (Figure 2a). Possible differential diagnoses were ventilated including abscesses, demyelinating disease and tumor recurrences. The MRI findings were without restricted diffusion, disfavoring an abscess diagnosis. There were no clinical symptoms of infection or demyelinating disease, and the lesions were interpreted as tumor relapse. No biopsy was taken. Chemotherapy was chosen as treatment modality with a palliative intent. The first procarbazine/CCNU/vincristine (PCV)-course was given in March 2004 (CCNU 120 mg/m^2^ Day 1 orally, procarbazine 100 mg/m^2^ Day 1 – 7 orally, vincristine 2 mg i.v. Day 1). MRI scan taken after 2 PCV-courses showed good remission with sparse contrast enhancement left in some of the previous lesions. After the 3rd course she developed severe bone marrow depression with thrombocytopenia (Trc 14 × 10^9^/l), leukocytopenia and anemia. MRI from August 2004 showed complete response from the chemotherapy without any pathological contrast enhanced areas left (Figure 2b). It was decided to administer additional chemotherapy, and due to the adverse effects from PCV the cytostatic agent was changed to temozolomide. The patient received 3 courses of temozolomide (reduced dose 150 mg/m^2^, 5/28 days schedule) during September to November 2004. 

MRI in December 2004 revealed postoperative changes and gliotic areas after previous lesions. Since then the patient has been to regular controls with MRI, and at the last control in February 2011 no relapse was observed. 

## Pathological findings 

**Figure 3.**
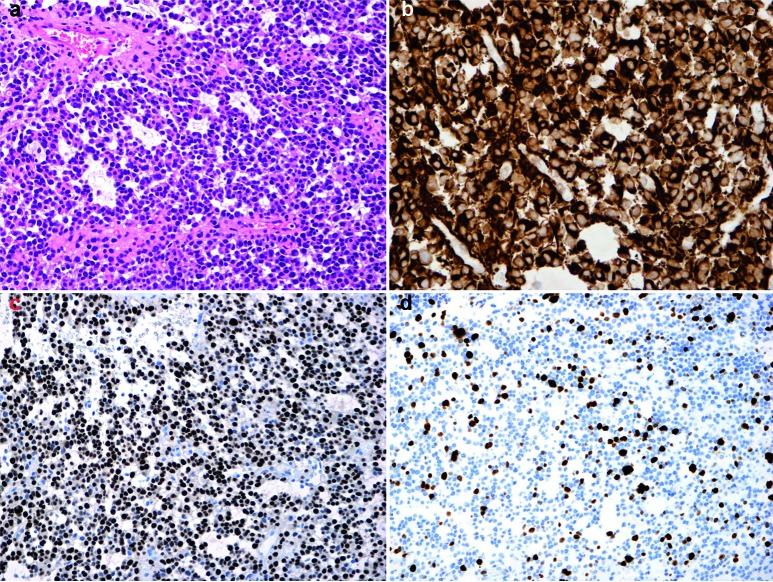
Histopathologic features of the tumor. (a) Cellular and nuclear atypia, hyalinized vessels, a papillary pattern and a pseudorosette (top left corner) with the typical stout processes (× 200). Positive immunostaining against GFAP (b) and p53 (c) (× 400). Several tumor cells with positive immunoreactivity for the proliferative marker Ki-67/MIB-1 (d) with an index of 10% (× 400).

The tumor was comprised of rather small-to-medium sized monotonous tumor cells with a reddish cytoplasm with eccentric round-oval hyperchromatic nuclei (gemistocyte like) without prominent nucleoli. The tumor cells revealed nuclear and cytological atypia, and were arranged in palisades or strings. Perivascular pseudorosettes were present in some areas. In other areas the tumor had a more solid growth and a microcystic background. Some blood vessels had hyalinized walls. No microvascular proliferation or necrosis was observed. 6 mitoses per 10 high power field (HPF) were counted. Results of immunohistochemical findings are listed in Table 1, and relevant microscopic images are shown in Figure 3. Electron microscopy showed bundles of intermediate filaments with no evidence of ependymal microvilli or neuronal differentiation. Fluorescence in situ hybridization (FISH) did neither show gene amplification of EGFR- or c-*erb*B-2 genes, nor polysomy of chromosome 7 and 17. 

## Discussion 

Our case was initially regarded as an anaplastic astrocytoma with predominant gemistocytic differentiation. Due to tumor localization, type of perivascular pseudorosettes, and abundant glial fibrillary acidic protein (GFAP)-positive eosinophilic epithelioid neoplastic astrocytes, it was subsequently considered to be consistent with an astroblastoma. Prominent hyalinization of the capillary network occurred only focally in this case. The disorganized astroblastoma pattern, cellular atypia, many mitoses, and high proliferative index assessed by Ki-67/MJB-1 and phospho-histone H3 (PHH3) advocated an anaplastic character, compatible with anaplastic astroblastoma. Other brain tumors with similar architectural features with pseudorosettes are ependymomas. However, the tumor cells appeared more epithelioid or cuboidal and lacked the typical round to oval nuclei with “salt and pepper” chromatin. Further, the pseudorosettes comprised of cellular processes shorter and broader than those of ependymomas. The paraffin sections did not show the periphery of the tumor; thus, we were not able to confirm the typically well defined demarcation of the astroblastoma histologically. MR, however, showed a well-demarcated tumor typical for astroblastoma. 

The positive immunostaining against GFAP and S-100 supports an astrocytic origin of this tumor. This was also confirmed by our electron microscopic examination which demonstrated intermediate filaments and no evidence of ependymal or neuronal differentiation. Positive immunoreaction of synaptophysin is in accordance with the reported nevronal elements in glial tumors [15]. 

Our patient underwent gross total resection of the tumor with adjuvant radiotherapy. Six months after surgery on a routine follow-up multiple cerebral lesions were found on MRI compatible with tumor recurrences. As they were located in both hemispheres and outside the irradiated area, so-called pseudo-progression was unlikely. It was then decided to administer chemotherapy with a palliative intent. No biopsies were taken. Five months later the lesions were turned into small lacunary areas surrounded by gliosis. 6.5 years after chemotherapy no recurrence has been recorded. The probable positive response for temozolomide may be due to the low level of the DNA repair enzyme *O* ^6^-methylguanine-DNA methyltransferase (MGMT), demonstrated by our negative immunostaining, as absence of this enzyme in gliomas is associated with increased chemosensitivity [16]. Reviewing the literature no treatment protocols for patients with astroblastoma are hitherto established, however, gross total resection appears as the treatment of choice [8, 9, 10]. For anaplastic forms adjuvant therapy (radiotherapy and/or chemotherapy) should be considered [8, 9, 10, 13]. As far as chemotherapy is concerned, various regimes have been tried out including cisplatin, cyclophosphamide, etoposide and vincristine [8, 13], however, during recent years temozolomide has gained much interest with promising effect [9], as in our case. 

EGFR, c-*erb*B-2 and c-*erb*B-3 have been shown to be involved in the development and growth of diffuse astrocytomas [17]. The overexpression of these receptors in our case supports similar growth mechanisms in astroblastomas. Furthermore, the positive p53 immunostaining corresponds to the important role mutated p53 plays in low-grade diffuse astrocytomas as well [18]. 

## Conclusion 

Astroblastoma is a rare primary brain tumor posing diagnostic and treatment challenges. The low incidence rate makes it difficult to conduct studies to examine tumor characteristics and effects of different treatment regimes. In general, this tumor has an unpredictable course with a tendency of recurrence. Total resection is reported to provide long time survival, however, adjuvant chemotherapy with temozolomide may be a treatment option for patient with high-grade tumors. 

**Table 1.** Results of immunohistochemical analyses.

Antigen	Results	Description	Clone	Dilution	Producer
GFAP	+++		polyclonal	1 : 2000	Dako^2^
S-100	+++		polyclonal	1 : 3000	Dako
CD56	+++		1B6	1 : 200	NovoCastra^3^
CD57	++		NK-1	1 : 50	NovoCastra
Ki-67/MIB-1 index	10%		MIB 1	1 : 200	Dako
PHH3	0,46%	10 mitosis per 10 HPF	polyclonal	1 : 2000	Millipore^4^
Ep-CAM	–		Ber-EP4	1 : 200	Dako
CK-AE1/AE3	–		AE1/AE3	1 : 100	Dako
CK20	–		Ks 20.8	1 : 200	Dako
CK5/6	–		D5/16B4	1 : 80	Dako
CK7	–		OV-TL 12/30	1 : 800	Dako
CKHMW	–		34BE12	1 : 150	Dako
EMA	–		E29	1 : 750	Dako
CD34	++	Staining of vessels	QBEnd/10	1 : 100	NovoCastra
CD31	+	Staining of vessels	JC/70A	1 : 50	Dako
Factor VIII	++	Staining of vessels	polyclonal	1 : 2000	Dako
NFP	–		2F11	1 : 1500	Dako
NSE	+		BBS/NC/VI-H14	1 : 200	Dako
Chromogranin A	–		polyclonal	1 : 4000	Dako
Synaptophysin	++		27G12	1 : 200	NovoCastra
Neu-N	–		A60	1 : 300	Millipore
b-amyloid	–		6F/30	1 : 50	Dako
E-cadherin	–		NCH-38	1 : 40	Dako
Gastrin	–		polyclonal	1 : 4000	Dako
Insulin	–		polyclonal	1 : 2000	Dako
Serotonin	–		5HT-H209	1 : 30	Dako
Glucagon	–		polyclonal	1 : 1200	Dako
Somatostatin	–		polyclonal	1 : 24000	Dako
Calcitonin	–		polyclonal	1 : 2000	Dako
CD20	–		L26	1 : 1000	Dako
CD3	+	Scattered in surrounding connective tissue	polyclonal	1 : 300	Dako
CD4	+	Scattered in surrounding connective tissue	4B12	1 : 150	NovoCastra
CD8	+	Scattered in surrounding connective tissue	C8/144B	1 : 200	Dako
CD68	+	Some activated microglia	KP1	1 : 3000	Dako
c-*erb*B-1**(EGFR)	++	Membranous staining	113	1 : 10	NovoCastra
c-*erb*B-2	++	Membranous staining	3B5	RTU^1^	Immunotech^5^
c-*erb*B-3	++	Membranous staining	RTJ1	1 : 10	NovoCastra
p53	+++	Nuclear staining	polyclonal	1 : 1500	NovoCastra
*O* ^6^-MGMT	–		MT23.2	1 : 200	Zymed^6^
Bcl-2	–		124	1 : 300	Dako
Estrogen	–		SP1	1 : 100	LabVision^7^
Progesterone	–		PGR-312	1 : 400	NovoCastra

^1^RTU = ready to use; ^2^Dako A/S, Glostrup, DK; ^3^NovoCastra Laboratories Ltd (NCL), Newcastle-upon-Tyne, UK; ^4^Millipore A/S, Oslo, NO; ^5^Immunotech SAS, Marseilles, FR; ^6^Zymed Laboratories Inc., San Francisco, CA, USA. Kindly performed by Dr. H. Broholm and H. Laursen at Department of Neuropathology, Rigshospitalet, Copenhagen, DK; ^7^Thermo Scientific/LabVision, Fremont, CA, USA; – negative staining; + weak staining; ++ moderate staining; +++ abundant staining. CKHMW = cytokeratin high molecular weight; EMA = epithelial membrane antigen; GFAP = glial fibrillary acidic protein; HPF = high power field; O6-MGMT = O6-methylguanine-DNA methyltransferase; NFP = neuron filament protein; NSE = neuron specific enolase; PHH3 = phospho-histone H3.
